# Morphio-Mania

**Published:** 1887-02-12

**Authors:** 


### MORPHIO-MANIA.

An Opium Eater.

EMILY NELSON was recently in custody at Bradford on a charge of theft. She could not be taken before the magistrates because she was in a condition of total imbecility. The poor woman was an opium eater. The police had no instructions to furnish opium or any other intoxicant to their prisoner, and so she lapsed into absolute incapacity for want of her accustomed dose. The prison surgeon, however, was equal to the occasion. He undertook to supply her for a few days with large doses of opium, in order to bring her into such a condition that she might be tried. The value of the quantity of opium required would be about two-and-sixpence per day.

Imbecility v. Drunken Intelligence.

A good many topics are suggested to the mind by this brief record. There was an Emily Nelson charged with theft, who could not be tried because she was an imbecile. By the aid of a powerful drug she could be restored to sanity and strength. She would then be quite a different Emily Nelson from her imbecile self. Which was the true Emily, the sane or the insane, the sober or the drunken? Which Emily was it that fell into theft and crime? Was it Emily sober and imbecile, or Emily drunk and full of opium and intellectual capacity? If the imbecile Emily committed the theft, then it was no theft, but the act of an irresponsible idiot. If the woman intoxicated, but made sane by the opium, then it was a drunken person who stole, and therefore also a person more or less irresponsible.

A Magistrate's Difficulty.

We do not envy a conscientious magistrate the duty thus laid upon him. The case of this woman is typical of many similar ones, and the magistrate's difficulty was exactly the problem which is presented to society by the great and growing increase of what, for want of a better name, may be called morphio-maniacs. Women and doctors —strange combination—are giving way to opium intoxication more than other persons, and in numbers that swell year by year. France leads the van in the dreadful procession, and Paris is easily first in France; Catholic France; atheistic France; clever, witty, and brilliant France. And what then? Is the subject worthy of anything more than a mere shrug of the shoulders and a passing sigh? Does it really matter whether a particular human being, or a score, or a thousand such beings take a brief and fitful delight in a poison-delirium for a few fleeting years and then die; or whether the same persons set themselves to earnest work for the same period and die sane like ordinary mortals? Does it really matter, O material philosopher? Is it worth while considering whether or not civilisation has any resources which can cope with so dire an evil? Or is it in a high philosophic sense not an evil at all, but a mere accident among the thousands of others which meet man on his journey from the first to the last unknown?

A Mad World!

The speculative philosopher may settle the speculative question as he will; the man of plain sense feels an imperative call to action. He wants to know why, in an age of political freedom and scientific progress, the world is growing more and more intolerable to all sorts and conditions of men? Why suicide is now considered by many, as it was in pagan Rome, a justifiable and even a meritorious act; why opium intoxication and other forms of drugging the senses into temporary excitement or oblivion, are for ever on the increase, and are spoken of in terms of pity and indulgence, rather than in those of disapproval and stern reprobation.

A Foul Blot and its Removal.

If there be anything in the principles which guide the conduct of modern society of a pernicious and dangerous character, the man possessed by the "enthusiasm of humanity" will seek to introduce sounder principles. If there be anything in the manners and customs of the people which tends to mental and moral feebleness and decay, he will endeavour to promote wiser habits and a sterner discipline. What is the cause? Where is the root of the evil? The effects we see. The gentle and virtuous woman reduced to loathsome idiotcy; the strong man degraded, wrecked, and ruined. This is a foul blot on our vaunted civilisation, an eating cancer preying on its life. Can history furnish us with any parallels whereby we may see the effect following the cause in a clear and unmistakable way? We think it can. Rome at the period of her decline and fall; France in the pre-revolution epoch—these show us how a luxurious civilisation led to a lowering of the standard of duty, to a vicious self-indulgence, to a speculative and practical atheism, which in turn produced weariness, disgust and hatred of life. Then came the tempter, in the shape of a potent drug or the swift steel, promising a visionary delight, a temporary forgetfulness, or an eternal sleep. Here lies the evil—why should we hesitate to name it? A weak self-indulgence, a low standard of duty, a practical atheism. Individual cases we may deal with as we can. It is possible to cure some of them, as the example of De Quincey shows—though hardly possible. But if the evil is to be dealt with as a whole, it is only by recognising the cause, going to the root of the matter, and teaching and practising a morality which has perfection for its aim, and the Divine approval for its reward. This, it seems to us, is the teaching alike of a true science, a rational philosophy, and a sober experience. These principles are part of the very constitution of man, and can no more be disregarded without injury and loss than can the physical demands of hunger and thirst.

